# Nutritional Challenges of Incretin-Based Obesity Management Medications: Implications for Clinical Practice

**DOI:** 10.1016/j.advnut.2025.100522

**Published:** 2025-09-18

**Authors:** Tair Ben-Porat, Shiri Sherf-Dagan, Marilou Côté, Cherie Josephine Miner, Assaf Buch

**Affiliations:** 1 School of Public Health, Faculty of Social Welfare and Health Sciences, University of Haifa, Haifa, Israel; 2 Department of Nutrition Sciences, School of Health Sciences, Ariel University, Ariel, Israel; 3 Department of Nutrition, Tel-Aviv Assuta Medical Center, Tel-Aviv, Israel; 4 Département des Fondements et Pratiques en Éducation, Faculté des Sciences de l’Éducation, Université Laval, Quebec City, Québec, Canada; 5 Centre Nutrition, Santé et Société (NUTRISS), INAF, Université Laval, Quebec City, Québec, Canada; 6 Institute of Endocrinology, Metabolism and Hypertension, Tel Aviv Sourasky Medical Center, Tel Aviv, Israel

**Keywords:** obesity, weight reduction, obesity management medications, GLP-1 and GIP receptor agonists, nutrition, eating behaviors, adverse effects, lean mass loss, behavioral interventions, lifestyle modifications

## Abstract

Several novel incretin-based obesity management medications (OMMs) have recently been approved for chronic weight management in adults with obesity or overweight. These agents have demonstrated substantial weight reduction effects alongside glucoregulatory and cardioprotective benefits. However, the use of incretin-based OMMs presents nutritional challenges that remain insufficiently addressed. These include side effects such as gastrointestinal disturbances and loss of lean body mass, which may compromise nutritional status, reduce energy expenditure, and heighten risk of rebound weight gain, sarcopenia, and frailty. Moreover, although these medications effectively suppress energy intake and reduce food quantity, they may also have unintended effects on diet quality, potentially influencing macronutrient distribution, ultraprocessed food consumption, risk of vitamin and mineral deficiencies, and disordered eating behaviors, which could undermine long-term weight maintenance and the cardiometabolic benefits achieved through these pharmacotherapy agents. Emerging evidence suggests that specific dietary and behavioral strategies, such as higher protein intake, resistance training, nutrient-dense eating patterns, and fostering adaptive eating behaviors, may help mitigate nutritional challenges and physiologic deterioration during significant weight reduction while also supporting cardiometabolic health maintenance. However, the application of these strategies as adjunct treatments alongside the new OMMs remains unclear. This narrative review summarizes the current literature on these issues and proposes dietary interventions and behavioral modification strategies aimed at mitigating the adverse effects that can be associated with incretin-based OMMs. These considerations are increasingly important given the expanding use of these medications, the degree of weight reduction they induce, and the implications for specific at-risk groups, including aging populations prone to muscle and functional decline and individuals with pre-existing conditions of nutritional deficiencies, chronic diseases, and disordered eating patterns.

**Statement of Significance**

This narrative review synthesizes emerging evidence on the unintended nutritional consequences of incretin-based obesity management medications and identifies key domains requiring clinical attention, including gastrointestinal side effects, lean body mass preservation, diet quality, micronutrient intake adequacy, and ongoing monitoring to identify and treat disordered eating behaviors. It offers actionable strategies to mitigate nutritional deterioration and support long-term treatment adherence, proposing targeted dietary and lifestyle modifications as essential adjuncts to preserve nutritional status and promote sustained health and well-being.

## Introduction

Obesity is a complex, chronic, progressive, and relapsing disease that constitutes a significant global public health challenge and is strongly linked to an increased prevalence of various chronic conditions, including coronary artery disease, type 2 diabetes, several types of cancer, physical disability, and a shortened lifespan [[1], [2], [3]]. At the population level, BMI is a common screening tool for obesity, but its accuracy at the individual level is limited as obesity is characterized by excess body fat and its related complications rather than body size alone [4]. The global prevalence of adult overweight and obesity has increased dramatically over the years, with ∼43% of adults having a BMI ≥25.0 kg/m^2^ (i.e., living with overweight or obesity), including 16% who are living with obesity (BMI ≥30 kg/m^2^) [5]. Accordingly, the overall economic impact associated with high rates of overweight and obesity is projected to rise from $1.96 trillion in 2020 to >$4 trillion by 2035, ultimately reducing global gross domestic product by 2.9% [6]. The first-line treatment for obesity continues to be lifestyle modification interventions; however, behavioral change programs face notable challenges, including low adherence rates and the common issue of weight recurrence over time [[7], [8], [9], [10]].

A recent groundbreaking advancement in obesity treatment is the development of incretin-based obesity management medications (OMMs) [1]. Historically, pharmacotherapy for obesity has been associated with concerns regarding both efficacy and safety, but recently approved incretin-based OMMs, such as liraglutide, semaglutide, and tirzepatide, have demonstrated significant weight reduction effects, along with glucoregulatory and cardioprotective properties and a high safety profile [1,4,[11], [12], [13], [14], [15]]. However, these medications also present notable nutritional and metabolic challenges that remain insufficiently addressed, including gastrointestinal (GI) side effects, fatigue, and appetite suppression [[8], [9], [10],^16^]. How these challenges translate into the preferred adjunct treatment approaches for OMMs remains unclear as there is a significant lack of studies in this area [[8], [9], [10],^16^]. Moreover, whereas clinical trials have primarily focused on the effect of incretin-based OMMs combined with lifestyle modifications on weight and cardiometabolic outcomes compared with a placebo with lifestyle modifications, a critical gap remains in understanding how to best structure, deliver, and integrate adjunct interventions, particularly nutritional interventions to optimize treatment success [9,10,17]. This narrative review aims to summarize current knowledge and highlight key priorities for future research, particularly regarding the development of effective adjunct behavioral and nutritional-focused strategies to support the optimization of health and nutritional status in individuals using incretin-based OMMs.

## Literature Search

A literature search of 3 electronic databases (PubMed, Cochrane Library, and Google Scholar) was performed as appropriate for narrative reviews. The main queries were the following: *1*) the impact of incretin-based OMMs on dietary intake, eating patterns, and nutritional status, as well as the underlying mechanisms; and *2*) the clinical strategies to mitigate nutrition-related side effects and complications associated with incretin-based OMM treatment. Accordingly, a combination of the following search terms was used: “obesity,” “weight loss,” “GLP 1 receptor agonists,” “incretin therapy,” “Semaglutide,” “Liraglutide,” “Tirzepatide,” “diet,” “nutrition,” “nutritional deficiencies,” “micronutrients,” “macronutrients,” “protein intake,” “body composition,” “muscle mass,” “lean mass,” “sarcopenia,” “maladaptive eating,” “eating behavior,” “eating disorder,” “side effect,” ‘adverse effect,” “gastrointestinal symptoms,” “nausea,” “vomiting,” “constipation,” “diarrhea,” “intervention,” “behavioral intervention,” “behavior change,” and “lifestyle modification.” The last of these searches was carried out on 1 April, 2025. The exclusion criteria included case reports and editorials, papers for which full text was not available or were not in the English language, and studies that focused on pediatrics. The reference list of the included articles obtained was manually searched for additional articles.

## Current Status of Knowledge

### The role of incretin-based OMM treatment

OMMs are suggested for long-term weight management for adults with overweight and obesity [11]. Several novel incretin-based OMMs, primarily analogs of glucagon-like peptide-1 (GLP-1) and glucose-dependent insulinotropic polypeptide (GIP), have recently been approved by regulatory authorities for chronic weight management in adults with obesity or overweight and ≥1 comorbidity (e.g., type 2 diabetes, hypertension, or dyslipidemia) [4,[11], [12], [13]]. A recent position statement from the European Association for the Study of Obesity further supports considering the use of these medications among individuals with a BMI ≥25 kg/m^2^ and a waist-to-height ratio >0.5, particularly when medical, functional, or psychological impairments or complications are present [18].

Incretin-based OMMs mimic naturally occurring enteropancreatic peptide hormones secreted postprandially, primarily GLP-1 (i.e., semaglutide and liraglutide), GLP-1 in combination with GIP (i.e., tirzepatide), or both GIP and glucagon (i.e., retatrutide) [1,[19], [20], [21], [22]]. The GLP-1 hormone is released from L cells in the small intestine and colon in response to food intake and binds to receptors on target organs, where it stimulates insulin secretion and inhibits glucagon release, slows gastric emptying, and increases satiety [1,4,[11], [12], [13],^19^,[20], [21], [22]]. Together with GIP and glucagon, these hormones complement each other by influencing fat breakdown and increasing energy expenditure [1,19]. The latest generation of incretin-based OMMs has demonstrated substantial weight reduction effects (a mean total weight reduction of 15%–24%), along with glucoregulatory and cardioprotective properties [4,[11], [12], [13]], positioning them as one of the most effective therapies available, potentially surpassing other nonsurgical weight reduction treatments [1,12,23,24]. Moreover, improvements in cardiometabolic risk factors (e.g., waist circumference and lipid levels) and a 20% reduction in major adverse cardiovascular events after treatment with semaglutide have been demonstrated [1]. Consequently, their expanding clinical application reflects both their efficacy and favorable safety profile, with additional therapies expected to gain regulatory approval in the coming years [25].

## Potential Challenges of Incretin-Based OMMs and Their Clinical Consequences

Although incretin-based OMMs achieve substantial weight reduction and offer glucoregulatory and cardioprotective benefits [4,[11], [12], [13]], they can also present notable challenges that remain insufficiently explored and addressed [9,10,26].

### Nonadherence and treatment discontinuation

Incretin-based OMMs are associated with barriers related to adherence, which in turn can impact their long-term effectiveness [27,28]. Although some demographic and clinical predictors of OMM discontinuation have been identified, underlying reasons remain underexplored and may include side effects, financial burden, or reluctance to continue long-term treatment after weight reduction goals are met [26,29]. Importantly, data on commonly used medications indicate that treatment discontinuation is related to weight recurrence [27,28]. For example, discontinuation of semaglutide 2.4 mg has been shown to result in weight recurrence of approximately two-thirds of the initial weight within 1 y [27].

### Gastrointestinal Side Effects

Incretin-based therapies are frequently linked to transient, mild-to-moderate GI side effects, primarily during the initiation and dose-escalation phases of treatment [1,30]. In particular, these include nausea (25%–44%), diarrhea (21%–30%), and constipation (11%–24%) [1] and likely result from direct effects on GI functions, such as altering gastric emptying, intestinal motility, and transit time, as well as central activation of GLP-1 receptors in the brain, which further suppresses gastric emptying [30,31]. The median duration of nausea, vomiting, and diarrhea is short and is generally <10 d, whereas constipation is often more persistent [30,32,33]. Although typically transient, aside from constipation, which may persist for longer [1,30], GI side effects can recur with each dose escalation and may affect treatment adherence, dietary and fluid intake, and patients’ mental well-being [34]. Moreover, when persistent, GI side effects can markedly reduce food intake, increasing the risk of nutritional deficiencies and muscle loss beyond the medication’s appetite-suppressing effects [1,9].

### Body composition alterations

There is a direct relationship between the degree of weight reduction and the percentage of lean body mass (LBM) loss [35,36]. To date, similar LBM-to-weight reduction ratios have been observed across dietary interventions, incretin-based OMM therapies, and metabolic bariatric surgery (MBS) despite differences in total weight reduction magnitudes, with a slightly steeper LBM decline noted during incretin-based OMM treatments [36]. Weight reductions achieved with incretin-based OMM therapies (i.e., typically averaging 15%–24% of total body weight) have been associated with an average absolute loss of ∼6 kg in LBM, representing roughly a 10% decrease from pretreatment LBM levels [35] and matching the extent of muscle deterioration commonly observed over 10 y of aging [35,37]. More specifically, semaglutide (2.4 mg) treatment is associated with a 13.9% reduction in LBM (∼6.9 kg) over 68 wk, whereas tirzepatide treatment results in a 10.9% reduction (∼6 kg) over 72 wk [36]. The loss of LBM during weight reduction therapy with incretin-based OMMs can also be expressed as its relative loss from the total weight reduction achieved, with a reported range of LBM losses of 40%–25% [36,38]. Accordingly, a key concern with the increasing use of incretin-based OMMs is the potential risk of sarcopenia, particularly sarcopenic obesity, due to substantial reductions in both weight and LBM, a condition that is known to increase risk of functional decline and frailty [9,36,[38], [39], [40]]. Although concerns about the risk of sarcopenia or sarcopenic obesity with incretin-based OMM therapies persist, current evidence remains inconclusive, as existing studies were not specifically designed to assess these outcomes and are limited by small, selective, and heterogeneous samples [9,35,36,38,39]. An additional concern related to reductions in LBM and muscle mass during incretin-based OMM use is that such losses may contribute to a decline in basal metabolic rate (BMR), which can not only predispose individuals to future weight recurrence but also hinder the maintenance of cardiometabolic improvements achieved through OMM therapy [[41], [42], [43]].

### Fatigue

Individuals treated with incretin-based OMMs are twice as likely to experience fatigue compared with those receiving a placebo, despite clinical improvements in parameters such as heart failure indices, insulin sensitivity, and functional performance, factors generally linked to enhanced muscle quality [1,36,38]. The underlying causes of fatigue in these patients remain unclear; however, this phenomenon and its implications warrant further investigation [1,36,38].

### Dietary patterns and eating behaviors

To date, limited research has examined the effect of incretin-based OMMs on nutrient intake, overall dietary patterns, and eating behavior trajectories [34,44]. Although recent studies in individuals receiving incretin-based therapy for obesity or diabetes report 16%–39% reduction in total caloric intake, few have specifically evaluated changes in diet quality, including the actual intake of specific macronutrients and micronutrients [44]. Among the limited studies available, some have observed an overall reduction in macronutrient intake, as well as decreased consumption of high-fat and sweet foods during medication treatment [[44], [45], [46], [47], [48]]. However, specific dietary changes, such as the relative intake of macronutrients and micronutrients, fiber, and fluid, remain insufficiently understood [17,[44], [45], [46], [47], [48]]. Data are also scarce regarding potential changes in timing and number of daily meals and snacks, consumption of ultraprocessed foods, and patterns of problematic eating behaviors [9]. Additionally, data on the association between the use of incretin-based OMMs and the development of eating disorders, as well as on treatment outcomes in patients with pre-existing eating disorders, remain limited [9,17,26,[48], [49], [50], [51]]. Although eating behavior changes, including both resolution and emergence of disordered eating patterns, have been extensively documented in patients who underwent MBS, such findings may not be directly applicable to incretin-based OMM users because of fundamental differences in treatment mechanisms [26,52]. Importantly, patients using incretin-based OMMs may be more receptive to adopting healthier eating patterns as they often report reduced cravings, a diminished urge to overeat, and a decrease in “food noise,” a term referring to the persistent and distressing thoughts about food and the compulsion to eat [34,51,53,54]. For example, a secondary analysis of a randomized controlled trial (RCT) explored the effects of liraglutide (3.0 mg/d) combined with intensive behavioral therapy (IBT) on eating behaviors and eating disorder psychopathology in individuals with obesity. After 24 wk, participants receiving liraglutide plus IBT reported greater reductions in dietary disinhibition, global eating disorder psychopathology, and concerns about shape and weight compared with those who received IBT alone, although the effect sizes were small. However, by 52 wk, these differences were no longer significant, suggesting the initial benefits of liraglutide may diminish over time [55]. Collectively, future studies are needed to further investigate the effect of incretin-based OMMs on disordered eating behaviors [9,17,[49], [50], [51]]. Moreover, a key question arises regarding the consequences of discontinuing these medications, particularly regarding the potential exacerbation, recurrence, or development of problematic eating behaviors and eating disorders, such as uncontrolled eating and binge eating disorder [9,18,34,51,53]. Table 1 summarizes the main challenges associated with incretin-based OMMs and their nutritional consequences, focusing on key areas that require clinical attention and further investigation.

**TABLE 1:** Key challenges associated with incretin-based OMMs and their potential nutritional and clinical consequences.

Main challenge	Proposed mechanisms	Potential consequences
Adherence issues and treatment discontinuation	Long-term injections; side effects; access issues; mismatch between expectations and actual outcomes	Weight recurrence; reduced long-term effectiveness; can be linked to relapse in disordered eating behaviors
GI side effects (e.g., nausea, vomiting, constipation)	Activation of GLP-1 receptors in the GI tract influences gastric emptying, intestinal motility, and transit time; some GI side effects, such as nausea, also involve direct activation of GLP-1 receptors in the brain, further contributing to delayed gastric emptying	Reduced appetite; poor nutrient intake; risk of dehydration and nutritional deficiencies
Appetite suppression and reduced food quantity	Central appetite regulation via GLP-1/GIP receptor activation in the hypothalamus, slows gastric emptying, and reduces motility	Risk of inadequate caloric and protein intake; difficulty meeting nutritional needs; potential for nutrient deficiencies
LBM loss	Rapid weight reduction, potentially exacerbated by insufficient protein intake and/or lack of resistance training	Sarcopenia risk; reduced strength and physical function; might elevate risk for future weight recurrence due to BMR decrease
Fatigue	Might be linked to energy restriction, rapid weight reduction, or micronutrient inadequacies	Decreased physical performance; impaired adherence to activity guidelines; risk of muscle catabolism
Altered diet quality and eating behaviors	Appetite suppression without dietary guidance and/or adjustments; changes in food preferences; food intolerances; persistence or emergence of maladaptive eating patterns	Increased intake of ultra-processed foods; poor macronutrient balance; disordered eating; reduced sustainability of weight reduction; compromised cardiometabolic outcomes, quality of life, and well-being

Abbreviations: BMR, basal metabolic rate; GI, gastrointestinal; GIP, glucose-dependent insulinotropic polypeptide; GLP-1, glucagon-like peptide-1; LBM, lean body mass; OMM, obesity management medication.

## Mitigating Nutritional Risks of Incretin-Based OMMs: Clinical Practice Implications

Regulatory authorities recommend using incretin-based OMMs in conjunction with a reduced-calorie diet and increased physical activity [1]. However, there is a notable lack of evidence-based guidance for clinicians on how to support patients in adopting healthier lifestyle habits to optimize health outcomes during treatment with incretin-based OMMs [[8], [9], [10], [11],^26^,^56^]. Importantly, drug trials for obesity typically compare active treatment to placebo, with both groups receiving the same basic lifestyle adjuvant treatment, making it impossible to assess the efficacy of lifestyle modification in augmenting drug therapy [17]. Given this genuine equipoise, determining whether drug plus lifestyle intervention offers a health advantage over drug plus placebo or standard care in well-designed and adequately powered clinical trials is essential [17]. However, such studies are currently lacking or are still in the early stages of conceptualization or recruitment [9,57]. Collectively, there is strong evidence that structured lifestyle modification programs improve health in patients living with obesity, but there is a lack of information on the impact of such interventions in the context of incretin-based OMMs and the unique challenges they pose for patients [9,10,16,17,58]. Importantly, behavioral and nutritional support frameworks from other intensive obesity therapies, such as MBS and very low-calorie diets (VLCDs), might offer transferable strategies to support incretin-based OMM users and help overcome the nutritional risks discussed in this review [[8], [9], [10]]. For example, MBS protocols emphasize increased protein intake, micronutrient supplementation and monitoring, and physical activity to counteract LBM loss and nutrient deficiencies, issues that may also be relevant to patients undergoing significant weight reduction with incretin-based OMMs [[8], [9], [10]].

### Diet quality and intake adequacy

Nutritional deficiencies are commonly seen in MBS and VLCD and may also occur in patients achieving significant weight reduction with incretin-based OMM therapies [8,9]. Drawing from evidence in MBS and VLCD studies, micronutrients recommended for monitoring during extensive weight reduction include vitamin B12, folic acid, thiamine, and fat-soluble vitamins (A, D, E, and K), as well as iron, copper, zinc, magnesium, and calcium [9]. Accordingly, some of the previous reviews on this topic have recommended considering the use of a daily multivitamin for patients treated with incretin-based OMMs, particularly for those at risk of undernutrition, low intake, or persistent GI symptoms, with referral for nutritional assessment and monitoring if malnutrition or nutritional deficiencies are suspected [1,9,26]. Nevertheless, these recommendations are primarily based on clinical reasoning and prior experience with substantial weight reduction interventions, yet they are supported by limited, if any, research specifically focused on incretin-based OMMs. A recently published cross-sectional study examined dietary intake in a small convenience sample (*n* = 69) of patients treated with GLP-1 analogs for ≥1 mo. Using food diaries and comparing intake to individual Dietary Reference Intake recommendations, the study found that participants consumed insufficient amounts of protein (<1–1.2 g/kg/d) and dietary fiber [59]. Additionally, they demonstrated inadequate intake of several micronutrients, such as iron, magnesium, calcium, and vitamins A, D, and E, which have also been flagged as potential deficiencies in other weight loss interventions, including VLCDs and MBS [59]. Regarding diet composition, no specific dietary pattern has shown clear superiority for sustained weight reduction, but healthy and nutrient-rich dietary patterns, such as the Mediterranean or plant-based diets, have been consistently associated with improved health outcomes, and promoting adherence through culturally and economically tailored choices seems essential [8,56,[60], [61], [62], [63]].

### LBM preservation

When targeting body composition alterations, particularly muscle loss and risk of sarcopenia during weight reduction induced by incretin-based OMM therapies, the evidence remains limited and fragmented, and only a few prospective or interventional studies have examined the effect of behavioral strategies on preserving muscle mass or mitigating sarcopenia risk in this context [1,9]. Recent reviews on this topic emphasize combining resistance and aerobic activity to attenuate LBM loss and improve muscle quality and cardiometabolic health in incretin-based OMM treatment [9,39]. Nevertheless, limited data are available on the combined effects of dietary interventions and supervised physical activity on these outcomes in patients using incretin-based OMMs [1,9]. In 1 RCT, ∼160 adults (aged 18–65 y) completed an 8-wk VLCD phase before being allocated into 1 of 4 52-wk intervention groups: placebo, liraglutide 3 mg, liraglutide 3 mg plus supervised aerobic training, or placebo plus supervised aerobic training, with an exercise target of 75–150 min of moderate-to-vigorous aerobic activity per week. The greatest improvements in glycemic control, metabolic markers, and body composition were observed in the liraglutide plus exercise group [58]. A follow-up observational study showed that participants who discontinued both liraglutide and exercise had less weight recurrence and experienced fewer adverse body composition changes than those who stopped liraglutide alone, highlighting the protective role of physical activity when combined with pharmacotherapy [58]. Findings from a previous meta-analysis indicating that diet alone leads to ∼25% LBM loss, which is reduced to 11% when combined with physical activity, may help inform adjunct behavioral strategies for incretin-based OMMs aimed at preserving LBM [64]. Another meta-analysis demonstrated that combining resistance training with diet reduced LBM loss by 93.5%, resulting in a net preservation of ∼1 kg of LBM compared with diet alone, without compromising fat mass loss [65]. Evidence from MBS also emphasizes the value of incorporating physical activity and adequate intake of protein, calcium, and vitamin D to mitigate bone and LBM loss during massive weight reduction [55,[66], [67], [68], [69], [70]]. In the BABS study, an open-label RCT, 220 premenopausal women and similarly aged men with severe obesity who underwent Roux-en-Y gastric bypass or sleeve gastrectomy received a preoperative vitamin D loading phase followed by postoperative supplementation with vitamin D (16,000 IU/wk), calcium (1000 mg/d), and protein (individually based, ranging from 35 to 60 g/d), along with a physical exercise regime. Two years after surgery, the intervention significantly slowed the loss of bone mineral density and LBM. However, the combined nature of the intervention limits the ability to determine the specific contribution of each component [67]. Several RCTs have also examined the effects of whey protein supplementation (20–27 g/d) as part of a protein-enriched diet (0.8–1.2 g/kg/d) during energy-restricted weight reduction (∼600–750 kcal/d deficit) in middle-aged and older adults over periods ranging from 14 d to 24 wk and found that whey protein effectively mitigated muscle mass loss and stimulated greater postprandial myofibrillar protein synthesis compared with equivalent doses of soy protein [[71], [72], [73]]. Although further research is needed to determine the precise daily protein requirements for individuals using incretin-based OMMs, several recent expert reviews and guidance from other weight reduction modalities support a recommended intake of 1.2–2.0 g/kg of body weight, reference weight (i.e., adjusted or ideal), or fat-free mass for most adults undergoing weight reduction with incretin-based OMM [9,34,36,37,39,74,75].

### GI side effects

Regarding the issue of GI side effects during OMM treatment, despite the prevalence of these symptoms, scientific literature on their associated nutritional risks and the effectiveness of dietary management remains limited [1,9]. Current published clinical recommendations are based largely on expert consensus and practical experience from incretin-based OMMs and other obesity medical treatments such as MBS, and these recommendations suggest strategies such as limiting fatty, fried, and sugar-laden foods to reduce overall GI side effects and support dietary quality, reducing portion sizes and fat intake to alleviate nausea, increasing dietary fiber and fluid intake to address constipation, and avoiding meals within 2 h of bedtime to minimize heartburn [1,9]. Additional guidance includes eating slowly, consuming smaller and more frequent meals, and avoiding meal skipping [9].

### Mental health and emotional well-being

Drawing on experience from other intensive obesity therapies, psychological challenges and problematic eating patterns should be proactively monitored and addressed as part of the adjunct behavioral support provided to patients using incretin-based OMMs [8,17,52,56,76]. First, before initiating therapy, it is important to establish realistic treatment expectations, including the anticipated magnitude of weight reduction associated with different agents and the variability in individual responses [8,34]. Second, as shown in other intensive obesity therapies, psychological challenges, such as depression and social distress, can significantly impact lifestyle behaviors and adherence [8,34]. Nevertheless, little research has examined how substantial weight reduction resulting from incretin-based OMMs may influence key aspects of psychosocial functioning, including eating behaviors, body image, interpersonal relationships, and overall quality of life [8,9,17,34]. Importantly, disordered eating should be carefully evaluated and monitored throughout treatment with incretin-based OMMs, particularly considering the potential for these medications to exacerbate restrained eating [51], a known maintaining factor in eating disorders [77]. As recently reviewed, incretin-based OMMs may offer potential psychiatric benefits because of their anxiolytic and antidepressant effects, as well as their ability to reduce alcohol consumption and binge eating behaviors; however, emerging concerns from postmarketing data regarding depressive symptoms and suicidal ideation highlight the importance of careful monitoring, particularly among patients with pre-existing mental health conditions [17,78,79]. In that regard, a recent meta-analysis of 27 RCTs involving 32,357 individuals concluded that incretin-based therapy for diabetes or obesity is unlikely to increase the already low incidence of suicide-related adverse events within the control setting of RCTs [80]. Nevertheless, continued monitoring is warranted to identify individuals who may be at risk as the use of these medications becomes more widespread and prolonged [80]. Collectively, individuals with pre-existing mental health conditions, poor body image, or a history of disordered eating (e.g., binge eating, restrictive patterns, or emotional eating) may be particularly vulnerable to psychological distress and maladaptive behaviors during rapid weight reduction or after discontinuing medication usage [9,10,18,78]. Ongoing mental health assessment, monitoring, and support are therefore essential components of comprehensive care to safeguard patient well-being and long-term mental health outcomes [8,9,34,78,80,81].

**FIGURE 1:**
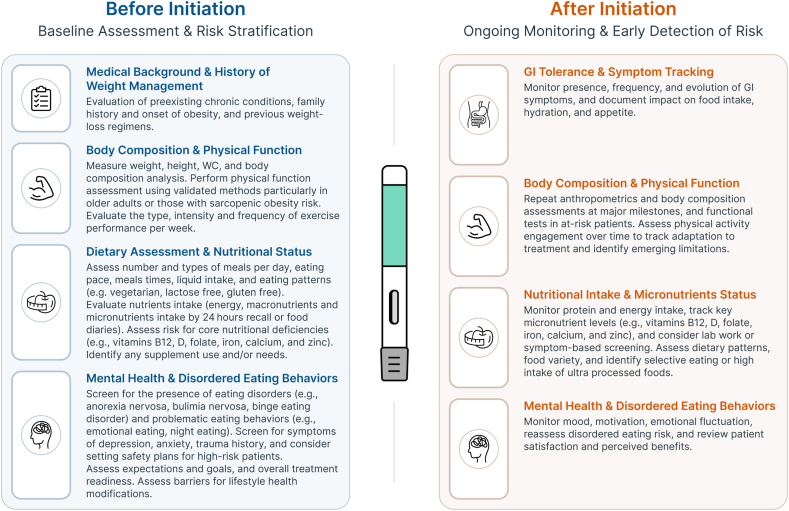
Nutritional and clinical monitoring timeline for incretin-based OMMs. GI, gastrointestinal; OMM, obesity management medication; WC, waist circumference.

Table 2 and Figure 1 summarize the topics discussed above, presenting key strategies and clinical recommendations to address nutritional challenges during incretin-based OMM treatment and offering a conceptual model for nutritional assessment and monitoring aligned with critical periods across the treatment trajectory, based on the existing literature in this area [8,9,17,26,[34], [35], [36],^39^,^52^,[74], [75], [76],[78], [79], [80],^82^]. Although these recommendations offer valuable guidance, their implementation is often limited by system-level barriers, such as time and resource constraints, availability of appropriate tools for accurate assessment and monitoring, limited access to dietitians, insufficient provider training in nutrition, and insufficient insurance coverage, highlighting the potential gap between evidence and real-world clinical capacity [26].

**TABLE 2:** Strategies to address incretin-based OMM nutritional challenges: clinical practice.

Issue	Clinical goal	Recommended strategy
GI side effects	Mitigate symptoms and preserve nutritional intake	• Follow recommended titration protocols strictly to minimize the severity and frequency of GI symptoms. Most GI issues occur during dose increases and can be mitigated by slowing the titration pace, if needed. • Eat smaller and more frequent meals (4–6 meals/d). • Avoid laying down after meals and aim to finish eating ≥2–3 h before bedtime. • Discourage meal skipping, which may exacerbate nausea or gastric discomfort. • Limit intake of fatty, fried, spicy, or ultra-processed foods, which may trigger GI symptoms. • Begin with easily digestible, bland foods (low-fat and low-fiber), especially during dose escalation. • Gradually reintroduce fiber-rich foods (e.g., fruits, vegetables, legumes) to promote bowel regularity and reduce constipation. • Promote mindful eating: slow pace, thorough chewing, and stopping at early satiety cues to avoid nausea or vomiting. • Ensure adequate fluid intake (∼1.5–2 L/d), and separate fluids from meals (30–60 min) to prevent early satiety/nausea. • Additional symptom-specific strategies: for nausea, cold bland foods may help; for constipation, gradual increase in fiber and hydration, physical activity, or stool softeners if needed; for diarrhea, reduce insoluble fiber temporarily and introduce soluble fiber sources (e.g., oats, bananas). • Persistent or severe symptoms warrant reassessment by the clinical team, including potential dose adjustment, nutritional consultation, or evaluation for intolerance.
LBM loss and sarcopenia risk	Prevent excessive loss of muscle mass and preserve physical function	• Integrate protein-focused nutrition and recommend daily protein intake of 1.2–2.0 g/kg of body weight, reference weight (i.e., adjusted or ideal), or fat-free mass, tailored to patient needs, to support muscle synthesis and slow muscle degradation. • Prioritize protein quality by encouraging intake of complete proteins (e.g., eggs, dairy, fish, lean meats, and soy) and high-quality plant-based sources (e.g., legumes and quinoa) to meet protein needs and support muscle synthesis. • Consider ONS in patients with reduced appetite, food aversions, or early satiety. • Consider distribution of protein intake by aiming for 25–30 g of high-quality protein per main meal (e.g., whey or leucine-rich sources) to stimulate muscle synthesis and counter anabolic resistance. • Incorporate regular resistance-based physical activity, ideally 2–3 times/wk, to maintain strength and functional capacity. • Combine resistance training with aerobic exercise to enhance LBM preservation and improve physical function. • Avoid extreme caloric restriction or prolonged undernutrition without supervision, especially in older adults, due to the heightened risk of sarcopenia and nutritional deficiencies.
Fatigue	Support energy levels, address underlying causes, and promote activity adherence	• Assess dietary intake to ensure caloric and protein adequacy during rapid weight reduction. • Tailor energy intake to prevent undernutrition, especially in patients with early satiety or food aversions. • Gradually reintroducing physical activity, starting with low-intensity aerobic and resistance-based movement as tolerated. • Further assessment of micronutrient deficiencies (e.g., vitamin B12 and iron) that can contribute to fatigue and/or functional tests to identify early signs of sarcopenia.
Altered nutritional intake, eating patterns, and diet quality	Maintain energy and nutrient intake, optimize diet composition, and prevent nutritional deficiencies	• Promote nutrient density by encouraging intake of whole, minimally processed foods rich in vitamins, minerals, fiber, and healthy fats (e.g., vegetables, fruits, legumes, nuts, whole grains, low-fat dairy, lean meat). • Ensure macronutrient balance by prioritizing high-quality protein and balanced carbohydrates and fats; avoid excessive restriction of any macronutrient group unless medically indicated. • Guide to energy-dense but healthy choices; use small, frequent meals and calorie-dense, nutrient-rich foods when intake is low due to early satiety. • Promote dietary patterns that support cardiometabolic health, such as the Mediterranean diet or other culturally appropriate, evidence-based approaches that emphasize whole foods, plant-forward eating, and minimal ultra-processed foods. • Support structured eating patterns despite reduced appetite or “food noise,” to ensure consistent nutrient intake and maintain metabolic balance. • Emphasize adequate fiber intake through whole grains, legumes, vegetables, and fruits to support glycemic control, bowel regularity, and satiety. Introduce gradually, particularly if GI tolerance is reduced during treatment initiation. • Address changing food preferences by monitoring for reduced variety or selective eating patterns. • Screen for micronutrient inadequacies by assessing key nutrients typically monitored during periods of rapid and extensive weight reduction (e.g., vitamin B12, folic acid, thiamine, fat-soluble vitamins A, D, E, and K, iron, copper, zinc, magnesium, and calcium), and provide supplementation based on individual evaluation. • Consider multivitamin supplementation in high-risk or low-intake individuals and those with persistent GI symptoms.
Psychological well-being and disordered eating behaviors	Ensure psychological stability, minimize risk of disordered eating development and/or recurrence	• Set realistic expectations by educating patients on the expected treatment course, possible fluctuations in mood or appetite, and the nonlinear nature of weight change. Set graduated therapy goals. • Promote body acceptance. • Integrate behavioral therapy and psychological counseling into the treatment plan to support mental health. • Consider the integration of behavior change techniques (e.g., goal setting, action planning, self-monitoring) to support long-term adherence and coping with dietary and activity changes. • Conduct routine psychological screening and ongoing monitoring for symptoms of anxiety, depression, trauma history, and disordered eating. • Implement maladaptive behavioral pattern prevention strategies by supporting patients in maintaining dietary structure, emotional regulation, and routine support.

Abbreviations: GI, gastrointestinal; LBM, lean body mass; OMM, obesity management medication; ONS, oral nutritional supplements.

## Conclusions

Incretin-based OMMs represent a paradigm shift in obesity treatment, offering substantial weight reduction along with glucoregulatory and cardioprotective benefits. However, their clinical use presents a unique set of nutritional challenges, including GI side effects, loss of LBM, and potential changes in eating behaviors and diet quality, that require careful management. These concerns seem particularly critical for vulnerable groups such as older adults, in whom rapid weight reduction may exacerbate sarcopenia and functional decline. Additional caution is warranted for individuals with pre-existing chronic conditions or comorbidities, including a history of psychiatric conditions and eating disorders, who may be especially susceptible to the unintended consequences of intensive weight reduction as well as medication discontinuation. Overall, integrating evidence-based nutritional and behavioral strategies tailored to the physiological and psychological demands of pharmacological treatment appears essential. These strategies include ensuring adequate total protein intake, distributing anabolic protein doses evenly across meals, incorporating physical activity and specifically resistance training to preserve muscle mass and strength, and addressing micronutrient requirements and risk of nutritional deficiencies. In parallel, structured behavioral support, individualized dietary counseling, and psychological monitoring are key to mitigating eating-related challenges, enhancing adherence, and promoting sustained long-term physical and mental health outcomes. Ultimately, promoting a patient-centered obesity management approach that prioritizes overall well-being, not just weight reduction as a primary outcome, seems essential. Moreover, implementing these strategies in everyday clinical settings will require overcoming systemic limitations such as gaps in provider education, challenges in patient access to clinical support, and the absence of consistent financial coverage for such services, as part of an integrated obesity care model. Although further prospective and mechanistic studies are needed to define best practices and optimize the structure of adjunct interventions, existing evidence underscores the importance of a proactive, multidisciplinary approach.

## Author contributions

The authors’ responsibilities were as follows – TBP, SSD, AB: conceptualized and designed this article; TBP, CJM, AB: performed the relevant literature search of the current evidence and search results screening; and all authors: responsible for writing the original draft and reviewing and editing the manuscript, read and approved the final manuscript.

## Data availability

Not Applicable.

## Funding

The authors received no financial support for the preparation of this article.

## Conflict of interest

TBP and SSD are members of the Communication and Development committee of the International Federation for the Surgery of Obesity and Metabolic Disorders, European chapter (IFSO-EC), which does not have any financial support or any relationship to the current article. All other authors have no conflicts of interest to declare. SSD and AB received research funding from Novo Nordisk. CJM is supported by the scholarship of Fullbright fellowship for excellence scholars. The supporting sources had no involvement in the writing of this paper or any restrictions regarding the submission of the article for publication.
